# Observational Study of Total Knee Arthroplasty in Aseptic Revision Surgery: Clinical Results

**DOI:** 10.1111/os.12593

**Published:** 2020-01-08

**Authors:** Gabriel Oliver, Luis Jaldin, Eric Camprubí, Guillermo Cortés

**Affiliations:** ^1^ Orthopaedics Department Head of Knee Unit Barcelona Spain; ^2^ Orthopaedics Department Hospital Universitario de Bellvitge Barcelona Spain

**Keywords:** Clinical results, Observational study, Total knee prosthesis revision

## Abstract

**Objective:**

To review the long‐term clinical results after revision surgery and the relationship between the different clinical variables involved with a failed total knee arthroplasty (TKA) and its evolution to provide a better understanding of the current treatment methods.

**Methods:**

The present study involved 89 subjects with a failed knee arthroplasty that ended up requiring revision surgery and component replacement between 2011 and 2015. The study included patients with pain remaining after TKA and indication from the knee unit surgeon to review the implant, without presenting with thromboembolic or neurological changes that could bias the results. The demographic data, surgical information, type of implant, and causes of failure were analyzed. The patients subjected to replacement surgery were specifically asked to fill out clinical and satisfaction questionnaires (Lysholm and KOOS). The mean follow‐up was 5.6 years (range, 3–11 years) and the analysis was divided into early revision (<5 years) and late revision (>5 years). The R statistical package version 3.2.5 for Windows was used, with significance less than 0.05 Cohort observational study.

**Results:**

The results indicated that implant revisions accounted for 5.57% of total primary implants, with a mean survival of 6 years for primary prosthesis failure. The mean revision surgery result on the Lysholm knee scoring scale was 68.73 out of 100 points. A better score was obtained for revisions undertaken on TKA with over 5 years' survival and there were no significant differences in terms of the type of implant used. The causes of TKA failure were aseptic loosening (77.38%), instability (9.52%), and painful prosthesis (13.10%). The results were statistically significant when isolated revisions were performed on one component. Rating worse on most of the questionnaire subscales.

**Conclusion:**

The clinical results were better in primary implant replacements with at least 5 years' survival. The replacement of only one of the components (tibial or femoral) provided worse clinical results than total replacement.

## Introduction

The increase in the number of total knee arthroplasties (TKA) is linked to a higher number of revisions. Given the demographic shift expected over the next 2 decades, patients are anticipated to undergo these procedures at younger ages compared with previous generations, such that those aged 65 years or younger will account for more than 55% of primary TKA^1^, ^2^, ^3^, ^4^. TKA is the treatment of choice for severe osteoarthritis of the knee joint.

It is widely known that knee replacement surgery provides good long‐term results^5^, ^6^. The constant technical changes in the history of TKA, as well as new instruments and materials, can explain the improvement in the clinical results. However, despite these advances, a significant revision rate remains. Compared with primary TKA, however, revision TKA have had less promising results, with survivorship as low as 60% over shorter periods^2^. In addition, recent studies have found an even higher degree of dissatisfaction and functional limitations among revision TKA patients than among primary TKA patients, 15% to 30% of whom are unhappy with their procedures^7^. These shortcomings of revision TKA are thought to result from several factors, including poor bone quality, insufficient bone stock, ligamentous instability, soft‐tissue incompetence, infection, malalignment, problems with extensor mechanisms, and substantial pain of uncertain etiology.

National Joint Registries provide a wealth of community‐based comparative data, including patient characteristics, implant factors, and surgical techniques. They highlight trends in the variation of outcomes while remaining sensitive to the impact of changing practice and allowing the identification of best practice as published by Delaunay^8^.

On examining the national records for different countries, the revision rate fluctuates between 5% and 10% of all arthroplasties performed^9^, ^10^. Patients who undergo revision TKA have an improved quality of life. Aseptic loosening is the most common reason for revision among the different national arthroplasty records, followed by infection, pain, stiffness, and instability. Revision TKA outcomes are thought to be related to several factors, including poor bone quality, bone defects, soft tissue instability and insufficiency, infection, poorly positioned components, extensor mechanism problems, and painful TKA with no apparent cause^2^. Clearly, TKA failure can pose a significant challenge for the health system, both in terms of the patient and funding the costs. Therefore, we designed this study with a clear objective to shed light on revision TKA outcomes to improve implant designs and surgical techniques.

Although there are several complex factors that can lead to worse outcomes with revision TKA, surgeons are expected to produce results equivalent to those of primary TKA. It is, therefore, important to highlight the objective and subjective outcomes of revision techniques to identify aspects which are susceptible to improvement. In this article, we supply a concise overview of revision TKA outcomes to help manufacturers, surgeons, and hospitals to improve on implant designs, surgical techniques, and guidelines for revision TKA.

Our study aims to assess the clinical results after revision surgery:

1. We review the evidence on four points: aseptic survivorship, functional outcomes, patient satisfaction, and quality of life.
2. The working hypothesis is that early revision surgery, prior to 5 years, presents worse long‐term clinical results.
3. The secondary objective is to analyze the impact on the final outcome when replacing just one of the implants (tibia *vs* femur).

## Materials and Methods

An observational study was undertaken of 89 patients that received a prosthetic knee replacement (89 implants) between 2011 and 2015. The mean follow‐up of the revision arthroplasty was 5.6 years (range, 3–11 years).

### *Data Collection*

A specifically designed knee unit database was used. It included general demographic variables such as age and gender, and other specific variables such as the cause of the primary prosthesis failure, the type of implant undertaken, surgical notes and incidences such as ligament instability, the loosening of one or all of the components, the replacement of one or all of the components, and the implant survival time, obtained from the surgical form and the clinical course^1^.

The inclusion criteria comprised: (i) patients with painful TKA and the knee unit surgeon's indication to review the implant, without presenting thromboembolic or neurological changes that could bias the results; (ii) every patient underwent a biopsy in the operating theatre using arthroscopy for subsequent microbiological analysis of the synovial and joint fluid samples, and to rule out infection; and (iii) the patient's ability to understand the instructions and answer the clinical questionnaires was also considered. This study's exclusion criteria were as follows: (i) septic type revisions (positive cultures for aerobic germs in at least two out of four samples, a positive anaerobic culture, mycotic infection, or tuberculosis) treated in a specific septic surgery unit in our orthopaedics department (study protocol of prosthetic infection with a complete blood test, bone scintigraphy with technetium 99 and marked leukocytes, and a joint biopsy); (ii) single compartment prosthesis revisions; (iii) second revisions; (iv) periprosthetic fractures; and (v) terminal patients.

Three expert surgeons from the knee unit performed the patient operations. They followed the antithrombotic prophylaxis protocol with enoxaparin 40 mg/24 h from the same night after the operation and an antibiotic prior to the intervention (cefazolin 2 g i.v.).

The different types of implants used in the revision were collected. They were all constrained prostheses (LEGION by Smith and Nephew, aMP revision by Wright, and Vanguard 360 by Biomet). The reviewed primary prostheses were posterior‐stabilized cemented implants. The patients subjected to replacement surgery were specifically requested to fill out the clinical and satisfaction questionnaires during 2016. Scales validated and presented in other publications were used, such as the Lysholm^11^, ^12^ score for evaluating knee function and the Knee Injury and Osteoarthritis Outcome Score (KOOS)^13^ questionnaire, including quality of life assessment. Every patient was examined by X‐ray before and after the operation, although that was not the objective of this study like in other published works^3^, ^14^.

### *Knee Injury and Osteoarthritis Outcome Score*

The KOOS questionnaire was developed in the 1990s as an instrument to assess the patient's opinion about their knee and associated problems. It consists of five subscales: pain, symptoms, function in daily living, function in sport and recreation, and knee‐related quality of life. Standardized answer options are included and each question is assigned a score from 0 to 4. A normalized score (100 indicating no symptoms and 0 indicating extreme symptoms) is calculated for each subscale.

### *Lysholm Knee Scoring System*

The Lysholm Scale is a patient‐reported outcome measure (PROM) to evaluate knee function and consists of eight items that measure: pain (25 points), instability (25), locking (15), swelling (10), limp (5), stair climbing (10), squatting (5), and need for support (5). The total score is the sum of each response to the eight questions, and may range from 0 to 100. Higher scores indicate a better outcome.

All subjects signed an informed consent agreement prior to their participation.

### *Statistical Analysis*

The nominal categorical variables are described by the number of cases, the percentage, and the number of missing values. The ordinal categorical variables and the continuous variables are described by the number of cases, the mean, the interquartile range, and the number of missing values.

The χ^2^‐test was used to compare two categorical variables. Student's *t*‐test or the Wilcoxon test was used to compare the means between the groups, based on the distribution of the continuous variable.

The TKA survival data up to 5 years and over 5 years was allocated for statistical analysis to evaluate the impact on the results.

The statistical significance was set at a <0.05 probability level. The R statistical package version 3.2.5 for Windows was used. The statistics department manager in our center's research foundation oversaw the analysis.

## Results

### *Demographic Characteristics and Follow Up*

A total of 89 patients were reviewed and demographic data was collected, such as age, gender, left/right side, and etiology (Table 1). During the study period from 2011 to 2015, 1595 TKA were performed, with 89 implants reviewed, meaning that the revisions accounted for 5.57% of all TKA. A total of 5 individuals could not be fully analyzed as not enough data was collected, so the final analysis was undertaken on 84 subjects. The failed primary TKA survival time ranged from 4 months to 25 years, with a mean of 6 years and a median of 4 years. When results were assessed in regard to gender, the differences were not significant. The etiology of failure of the primary total knee was aseptic loosening in 65 subjects (77.38%), instability in 8 (9.52%), and pain/stiffness in 11 (13.10%).

**Table 1:** Demographic characteristics

Variables	
Age (years)	
Mean (SD)	71.94 (9,07)
Median (IQR)	73.50 (66.75–78.00)
Gender [cases (%)]	
Female	63 (75.00)
Male	21 (25.00)
Side [cases(%)]	
Right	41 (48.81)
Left	43 (51.19)
Etiology [cases(%)]	
Loosening	65 (77.38)
Instability	8 (9.52)
Painful/stiff TKA	11 (13.10)

IQR, interquartile range; SD, standard deviation; TKA, total knee arthroplasty.

### *General Results*

An anterior longitudinal approach of the knee was performed with a subsequent paratendinous incision. No tibial anterior tubercle osteotomy or quadriceps snip was used.

The operation time was 100–145 min, with an average of 122.35 ± 17.78 min. Intraoperative bleeding was 300–400 mL, with an average of 324 ± 67 mL.

### *Type of Prosthesis and Failure*

#### *Intraoperative Results*

Table 2 shows the characteristics of the prostheses used at the time of the revision: 63 patients only presented a loose tibial component, while all components were involved in 19 patients (tibial and femoral). Only 2 patients had a loose femoral component in isolation. The analysis also looked at the revisions undertaken involving replacing just one of the components in isolation compared to replacement surgery for all the components (Table 2). One case involved only replacing the patellar component (secondary prosthesis). Table 3 shows the clinical results from the KOOS and Lysholm questionnaires. The mean quality of life score was 52.30 (ranging from 0 to 100, where 0 is the worst possible symptoms and 100 indicates no symptoms) (Table 3).

**Table 2:** Prosthesis characteristics [cases (%)]

Variables	
Type	
CCK	35 (41.67)
Constrained	15 (17.86)
Ultra‐congruent	12 (14.29)
TCPR	20 (23.81)
FCPR	1 (1.19)
PSP	1 (1.19)

TCPR, tibial component partial replacement.

FCPR, femoral component partial replacement

PSP, patellar secondary prosthesis.

**Table 3:** Clinical and quality of life questionnaires

Variables	
Lysholm (<65 poor, 66–83 mild, 84–90 good, > 90 excellent)	
Mean (SD)	68.73 (18.90)
Median (range)	70.50 (53.00–85.00)
KOOS symptoms	
Mean (SD)	84.94 (15.37)
Median (range)	89.29 (78.57–96.43)
KOOS pain	
Mean (SD)	76.07 (17.95)
Median (range)	77.78 (63.89–92.50)
KOOS function	
Mean (SD)	76.84 (16.56)
Median (range)	77.94 (65.44–91.09)
KOOS sport	
Mean (SD)	34.77 (17.11)
Median (range)	30.00 (25.00–45.00)
KOOS quality of life	
Mean (SD)	52.30 (28.32)
Median (range)	56.25 (25.00–78.12)

Knee Injury and Osteoarthritis Outcome Score (KOOS), scores range from 0 to 100 with a score of 0 indicating the worst possible knee symptoms and 100 indicating no knee symptoms

**Figure 1:**
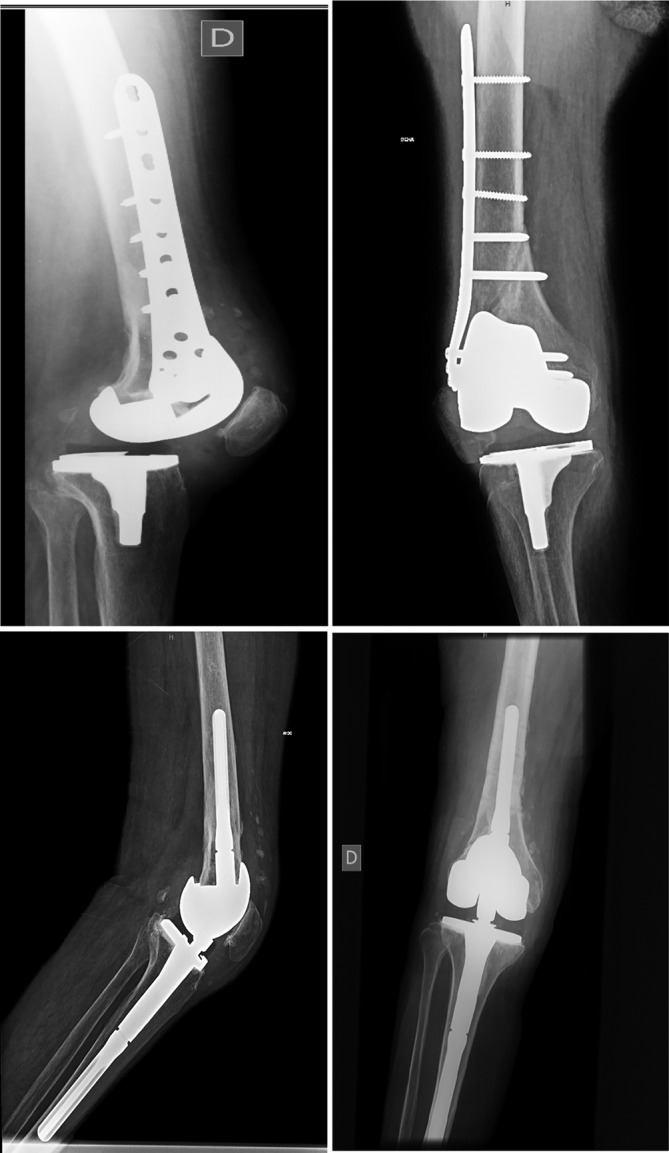
Revision surgery of a primary total knee with instability after femoral fracture in a patient of 75 years old. The fracture was initially reduced and fixed with a locking plate. A rotating hinge implant was needed to restore knee function.

**Figure 2:**
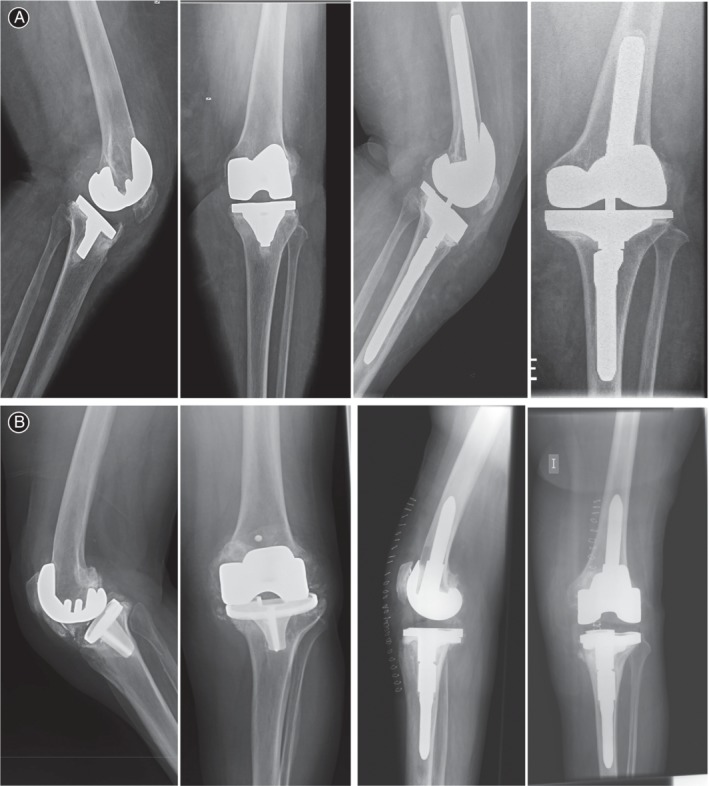
(A) 55‐year‐old obese women, with painful total knee replacement after 2 years’ follow‐up. The image shows a tibial component loosening with subsidence. A constrained condylar knee was used to reconstruct the joint line. The knee function was good after surgery. (B) Male patient, 68 years old. Images show a loosening of both tibial and femoral components. A very important collapse of the tibial component was treated with a constrained cemented prosthesis with good final outcome.

Revision surgery for the TKA cases of more or less than 5 years' survival involved 40 revisions due to mechanical loosening prior to 5 years, with 25 after 5 years, 6 due to instability prior to 5 years, and 2 after 5 years; there were 8 TKA revisions due to pain/stiffness prior to 5 years and 3 after 5 years (Figs 1 and 2).

#### *Clinical Results and Functional Evaluation*

The clinical and quality of life questionnaires obtained higher results for the revisions undertaken after 5 years, with a significant value for quality of life 66.77 (SD = 32.06, *P* = 0.025) and the KOOS pain 84.35 (SD = 17.71, *P* = 0.045) (Table 4).

**Table 4:** Clinical and quality of life questionnaires by revision time[mean (SD)]

Variables	<5 years, “*n*” = 54	>5 years, “*n*” = 30	*P*‐value*
Lysholm	65.10 (17.25)	76.50 (20.55)	0.0615
KOOS symptoms	82.85 (15.65)	89.77 (14.11)	0.1784
KOOS pain	72.49 (17.11)	84.35 (17.71)	0.0451
KOOS functional	73.92 (15.86)	83.58 (16.78)	0.0787
KOOS sport	33.17 (14.05)	38.46 (22.95)	0.3576
KOOS quality of life	46.02 (24.53)	66.77 (32.06)	0.0255

* *t*‐test with equal variances. KOOS, Knee Injury and Osteoarthritis Outcome Score; SD, standard deviation.

The correlation between the clinical results and the cause of failure (instability, mechanical loosening, and painful prosthesis) was not significant. There were no significant differences with respect to the type of implant used either (Table 5).

**Table 5:** Clinical and quality of life questionnaires by implant type. Non‐significant results between implants[mean (SD)]

Variables	Ultra‐congruent, “*n*” = 12	Constrained, “*n*” = 15	CCK, “*n*” = 35	*P*‐value*
Lysholm	62.80 (12.86)	65.29 (24.27)	79.44 (12.86)	0.955
KOOS symptoms	80.00 (21.81)	85.59 (19.18)	90.12 (8.43)	0.625
KOOS pain	70.56 (23.78)	80.06 (20.06)	82.98 (13.72)	0.434
KOOS function	76.18 (20.04)	79.81 (20.67)	82.70 (13.69)	0.780
KOOS sport	40.00 (15.41)	37.86 (26.44)	41.47 (14.00)	0.797
KOOS quality of life	41.25 (29.51)	59.71 (35.58)	64.68 (24.60)	0.355

* Variance analysis ultra‐congruent, aMP (Wright); Constrained, Vanguard (Biomet); CCK, Legion (Smith nephew). KOOS, Knee Injury and Osteoarthritis Outcome Score; SD, standard deviation.

There were significant differences between the results for the revision surgery in the one component (tibial or femoral implant) group and the revision surgery in the all components (tibial and femoral implant replacement) group for most of the KOOS clinical and quality of life values, with better results for the revision of all components group (Table 6).

**Table 6:** Clinical and quality of life questionnaires comparing revisions of one component with revisions of all components [mean (SD)]

Variables	All, “*n*” = 62	One in isolation, “*n*” = 22	*P*‐value*
Lysholm	73.37 (18.63)	58.79 (15.84)	**0.0153**
KOOS symptoms	87.28 (14.23)	80.10 (17.03)	0.1536
KOOS pain	80.13 (17.20)	67.66 (17.04)	**0.0309**
KOOS functional	80.87 (16.19)	68.49 (14.50)	**0.0197**
KOOS sport	40.34 (17.27)	23.21 (9.53)	**0.0013**
KOOS Q. of life	59.44 (28.59)	37.50 (21.93)	**0.0154**

* *t*‐test with equal variances. KOOS, Knee Injury and Osteoarthritis Outcome Score; SD, standard deviation.

### *Complications*

In terms of complications, there was 1 case of deep vein thrombosis treated with anticoagulant dose of subcutaneous enoxaparin for 6 weeks, 3 patients with skin involvement that resolved with rest and anti‐inflammatory treatment, 1 case of patellar instability that was revised with a soft tissue balancing procedure, and 1 case of wound dehiscence. There were no cases of infection during the study period.

## Discussion

Total knee arthroplasty results are currently satisfactory^1^, but there is still a proportion of patients that experience poor functional and satisfaction outcomes, which lead to considerable socioeconomic problems. Knee arthroplasty activity records provide us with data that can help to predict which patients may experience more problems and what decisions can be taken to improve our revision surgery results.

This study highlights that the worst clinical outcome occurs when only one prosthetic component is reviewed, which also means it is possible to preserve the fixed part of the prosthesis, causing less morbidity during the second surgery and avoiding a high financial cost^2^. The current availability of modular implants as part of a system encompassing the primary prosthesis for the revision prosthesis means that made to measure replacement is possible. There were 62 revision operations that involved replacing all the components. All components were loose in only 19 cases. The surgeon's decision to extend the revision to every component derived from having to constrict the implant more or from considering that a fixed component may be incorrectly positioned. Our study has expanded the knowledge on an issue that has been analyzed little in the published literature and has provided very significant results, with complete revisions of all the components evaluated higher than reviewing components in isolation. The Lysholm scale, and the pain, function, and satisfaction subscales of the KOOS questionnaire satisfaction results were significantly better for complete revisions. This could be explained by the fact that one loose component may not be the only cause of the failure, but rather that the remaining components may be incorrectly positioned, despite not being loose. This can lead to incorrect ligament balance or even using an incorrect size.

Our study results with respect to revision percentage are similar to most of the published series, at under 10%^14^, ^15^, ^16^, ^17^. The Lysholm scale and KOOS clinical and satisfaction outcome was acceptable, supporting the surgery indication criteria. The results show that the main cause was aseptic loosening, followed by instability, and painful prosthesis, with the loosening distributed evenly across the follow up period. This was not the case for instability and painful prosthesis, which mainly presented within the first 5 years of primary prosthesis survival, as opposed to other series in which they were not as limited to that period^1^, ^9^, ^17^, ^18^, ^19^. Instability is the cause of a poor clinical outcome that means second operations are required in the short term, as discussed by Song *et al*.^19^, with the mean revision of the first implant instabilities after 3.5 years of survival. With respect to painful prostheses of no apparent cause appearing in all the published series as one of the causes of arthroplasty failure, explaining the true expectations of this type of surgery properly to patients could lead to an improvement and reduce its frequency^18^.

The clinical results, the Lysholm results, and all the KOOS questionnaire subscales show worse scores for the revisions undertaken prior to 5 years (i.e. early)^18^, ^20^. In the case of pain, the best score is statistically significant when the revision was undertaken later (Table 4). We believe that a cause that leads to early implant revision leads to a more serious mechanical change in the joint compared to one that occurs later or more progressively. In revision surgery, the magnitude of the cause will make restoring the original biomechanics and functionality somewhat harder, resulting in a worse final score.

Many factors lead to implants failing, including the types of material, fixation, polyethylene, and technique used (surgeon‐dependent)^8^, ^21^. The latter could have some effect on the poor evolution of implants over the short term when instabilities and painful prostheses typically occur.

Our department has mainly used constrained type revision prosthesis, following the surgeon's criteria in evaluating each case, with a more constricted implant used with major bone defects and instabilities. No significantly different clinical or satisfaction results were obtained^13^, ^22^.

The limitations of this study are well understood and inherent to those of an observational study that collects detailed data but cannot target a specific aspect. Another difficulty comes from treating this patient type, most of which are elderly and have multiple diseases^23^. Finally, three types of implants are used for revisions, which the surgeon selects on a case by case basis. However, when detailing the individual results, the impact of this data declines, as there are no significant differences between them (Table 5). Similar results were published by Hwang *et al*.^24^.

We would like to conclude by highlighting that mechanical loosening is the main cause of revision. Primary prosthesis revisions undertaken after at least 5 years show a better result than those that failed prior to 5 years. When compared to total replacements, the revision of only one component, always a difficult decision in everyday clinical practice, requires careful analysis and consideration to avoid worse results and potentially having to operate again in the future.
